# Rethinking the portrayal of deaf characters in children's picture books

**DOI:** 10.3389/fpsyg.2013.00889

**Published:** 2013-11-27

**Authors:** Debbie B. Golos, Annie M. Moses

**Affiliations:** ^1^Department of Communication Disorders and Deaf Education, Utah State UniversityLogan, UT, USA; ^2^Department of Education and Allied Studies, John Carroll University, University HeightsOH, USA

**Keywords:** deaf, culture, portrayal, children's picture books, deaf gain

Children's literature provides the opportunity for children to see both representations of themselves and of others in print and in pictures. Not only can engaging with picture books develop key literacy and language skills and a love of reading, but reading (and/or being read) high quality books also has the potential to foster an appreciation and respect for oneself and others. However, sometimes the diversity that exists in the real world is not represented well—or at all—in books created for young children (Mendoza and Reese, 2001; Kama, 2004). In this article we will discuss the lack of, and critical need for, children's books that portray Deaf^1^ characters from a cultural perspective. We will also provide examples, from a sampling of children's picture books, of messages about deafness and deaf characters in text and illustrations (e.g., Golos and Moses, 2011; Golos et al., 2012). Finally, we will highlight the implications for both hearing and d/Deaf children as well as parents and educators.

Typically, when people think about deafness, they think about a person with a disability, that is, someone who is unable to do something (i.e., hear). This represents a pathological perspective of deafness, which entails viewing deafness as a disability or as a condition that needs to be fixed with medical intervention (i.e., cochlear implants or hearing aids; e.g., Lane, 1992; Lane et al., 1996; Padden and Humphries, 2006). Deaf studies scholars suggest an alternative view of deafness from a cultural perspective, in which the deaf person is seen a member of a minority population, the Deaf community, rich with their own language (i.e., American Sign Language or ASL) and culture (i.e., Deaf culture) (e.g., Lane et al., 1996).

## Perspectives of deafness and deaf people in literature

Prior to 1990, when researchers examined portrayal of characters with disabilities in media, deafness, similar to other disabilities, was stereotyped as a person, “needing to be fixed,” “isolated,” “angry,” “in danger,” and/or “unable to function in daily life” (e.g., Carlisle, 1998). It has only been recently that some have begun to *compare* portrayals of deafness for pathological vs. cultural perspectives. Although there has been an increasing awareness of Deaf culture, evidence from empirical studies suggests that the pathological perspective prevails (Golos, 2010; Golos and Moses, 2011; Golos et al., 2012). For example, we content analyzed the text and illustrations of 20 children's picture books published after 1990 and that included a main character who was deaf (Golos and Moses, 2011; Golos et al., 2012). Results showed that the majority of messages related a pathological perspective of deafness (71% of messages in the text and 93% of the messages in the illustrations).

Because of the prevalence of messages reflecting the pathological model of deafness, young readers may be more likely to experience books about deaf characters attending public schools and being mainstreamed with hearing children, as well as struggles they may face in such settings. For example, more often they may learn about characters like Oliver who cannot hear and, as a result, is isolated until he receives his hearing aid (Riski and Klakow, 2001).

Although cultural portrayals exist in children's literature, characters like Oliver dominate the literature. We found that the most common pathologically-oriented messages included references to fixing the character's deafness and having a greater ability to function or an improved quality of life after receiving amplification (e.g., hearing aid or cochlear implant) (Golos and Moses, 2011; Golos et al., 2012).

Another recurring message involved the deaf child in danger. For example, in one picture book, there is concern that a deaf character might get hit by a car because the character could not hear (Roth, 2000). In another story, an elementary-aged deaf child gets upset and runs into an elevator, does not know how to use it and gets stuck. Other characters are concerned because the boy cannot hear their verbal instructions (Watkins, 1993). Similar to the disability literature, these, and other similar messages found in the sample, perpetuate a stereotype of a deaf child as needing to be fixed or needing help to function in everyday situations (Carlisle, 1998; Golos and Moses, 2011; Golos et al., 2012).

Although the sample was small, there were very few cultural messages found in the text and illustrations of the 20 picture books analyzed. One of the few examples comes from the *Moses* series, in which the deaf character, Moses, attends a school for the deaf and everyone signs (including his family). When his mother asks Moses how his first day of school was he responds, “I have 10 classmates… and all ten are my friends” (Millman, 2000, p. 27). Additional examples included aspects of the Deaf community (e.g., attending a Deaf theater production), or Deaf characters using flashing lights, and other *cultural* technologies used in everyday situations (i.e., without *medical* intervention) (e.g., Millman, 2000; Tildes, 2006).

However, it should be noted that books conveying more of a pathological perspective also contained some positive messages. For example, some books included messages related to positive relationships between the deaf child and his/her parent(s). In *Moonbird* (Dunbar, 2007), the parents learn the importance of communication and valuing their deaf child for who he is, and in *Dad and Me in the Morning* (Larkin, 1994), a hearing father and his deaf child bond while watching the sunrise. Moments such as these represent the critical connection between family members and their deaf child.

## Implications regarding perspectives on deafness and deaf people in picture books

One reason the pathological perspective dominates may be due to an increase in medical technology, such as cochlear implants. As a result, deaf children are increasingly mainstreamed into general education settings or placed in self-contained classrooms. However, in these settings, a deaf child often has limited interactions with other deaf children and/or Deaf adults, may not be taught or encouraged to use ASL and the classrooms often lack staff and/or resources to teach about Deaf culture.

Recent evidence suggests that deaf children, regardless of use of amplification, benefit from exposure to visual language such as ASL (e.g., Mayberry, 2007, 2010) and exposure to culturally Deaf role models (e.g., Holcomb, 1997). Deaf community members and national organizations for the Deaf (http://www.nad.org/issues/education/k-12/position-statement-schools-deaf) support schools for the deaf that offer access to ASL and Deaf culture as the preferred setting for most deaf children. However, for the increasing number of children not attending these schools, there may be a greater need for positive Deaf role models and positive messages about ASL. Utilizing children's picture books is one way to do so. They also can offer the hearing population messages to promote understanding and respect for d/Deaf people. However, to do so, they must contain accurate information about the Deaf community and Deaf culture.

Ultimately, picture books could have considerable impact on children's perceptions of themselves and others. If hearing children read (or are read) picture books that present a pathological view, they may learn that deafness is a disability, that a deaf person struggles in daily life, and that medical “fixes” are necessary in order for a deaf person to be happy, able to function, and be accepted. For the Deaf child, such depictions may cultivate a sense of inadequacy and/or low sense of self regarding who they are and what they can do. She/he may also feel the need to be “fixed.”

If, on the other hand, picture books portray a cultural perspective of Deaf people, then deaf and hearing children could learn that Deaf culture exists and thrives and learn more about the language, history and values that are distinct from the hearing population. Through exposure and discussion about picture book messages, the hearing population could even serve as advocates for preserving the Deaf child's language and culture. Deaf readers may see that, like the main character in the *Moses* series, they can go to a school where Deaf teachers use sign language (e.g., ASL) and can learn to enjoy the arts, athletics, and academics utilizing their strengths in a visual language (e.g., ASL) and in a visual environment. This may promote a feeling of pride and help develop a strong sense of self.

## Future directions

Too often, being different from the majority is depicted negatively. Deaf studies scholars have coined the term “Deaf Gain” as a way of looking at the Deaf community from a perspective of benefit rather than loss (Bauman and Murray, 2009). That is, viewing deaf individuals as providing a unique to contribution to his/her community and broader society. Most notable are the visual “ways of being” (Bahan, 2009), including communicating through a visual language (e.g., ASL) and designing visual environments (lots of light, seats arranged in a way that everyone can see one another, etc.).

To help promote the concept of “Deaf Gain” in children's picture books, we encourage parents and teachers to incorporate books with cultural messages into their classrooms, and discuss these cultural messages. Recent examples, in addition to the *Moses* series, include new eBooks from Deaf authors/illustrators such as the *Zoey Goes* series. According to their website, the goal of their eBooks is for deaf children to “see themselves” and for hearing children to “learn about a linguistic minority” (zoeygoes.com). Parents and teachers can capitalize on these messages by discussing with children (hearing or deaf) how deaf people benefit from and access visual ways of being, such as using a light to get their dogs attention instead of calling him. However, there remains a need for high quality literature that incorporates more of these types of messages.

When pathological messages *are* present, educators should take the time to discuss them. For example, after reading the book about the deaf boy who gets stuck in the elevator, adults might ask children if they think that situation is realistic. Or, they might present examples of how all children can use visual strategies (that deaf individuals use) to do things, such as cross the street. Finally, children can learn that all deaf children, regardless of their use of medical technology or background can have friends, have skills and talents to share with others, and can lead meaningful lives.

## Future research

As an area for future research, we suggest that children's picture books be examined for their quality. After examining some of the storylines and themes of children's picture books with deaf characters, one might question whether these stories would have been published if the characters were not deaf. For instance, would a story be published about an elementary-aged hearing child who did not know how to use an elevator? Although an empirical examination is needed to examine the quality of current literature, teachers should review storylines and messages in books before choosing to incorporate them in their classrooms. The ultimate goal is to choose books that are worthy of children's time and attention that help develop their early literacy skills, and that provide important and accurate information about diverse populations.

## Conclusion

Children's picture books hold the promise to promote awareness and appreciation. Yet in order to do this, diverse populations should be depicted positively and accurately. Picture books can portray “Deaf Gain” through images, fictional stories, biographies and other genres that portray the unique qualities and advantages of visual ways of being rather than the loss of hearing. However, even when alternate messages are present these topics should still be discussed with children so they develop an awareness and appreciation in hearing readers as well as pride and connection to Deaf culture and the Deaf Community in d/Deaf readers.

Just as our own favorite picture book from childhood stays with us through the years, so may the other characters and messages portrayed in picture books that display diverse people and diverse ways of being. Picture books have the potential to influence our thoughts, perceptions and identity, and choosing and discussing picture books for young children provides daily opportunities to inspire and enlighten. Ideally, books chosen for children should foster these positive outcomes creating both an immediate and lasting positive impression of themselves and others.

## Footnotes

^1^For the purpose of this paper, we define “d” deaf as someone with a hearing loss and “D” Deaf as someone who identifies themselves with the Deaf community.
